# Familial *DMRT1*-related non-obstructive azoospermia: a case report

**DOI:** 10.1007/s10815-024-03250-2

**Published:** 2024-09-11

**Authors:** Giulia Severi, Enrico Ambrosini, Luca Caramanna, Luigi Monti, Pamela Magini, Giovanni Innella

**Affiliations:** 1 Medical Genetics Unit, IRCCS Azienda Ospedaliero-Universitaria di Bologna, Bologna, Italy; 2 Medical Genetics Unit, University Hospital of Parma, Parma, Italy; 3 Department of Medical and Surgical Sciences (DIMEC), University of Bologna, Bologna, Italy

**Keywords:** *DMRT1*, Non-obstructive azoospermia, Infertility, Reproductive medicine, Varicocele, Case report

## Abstract

**Purpose:**

To report an exceptional case of male-to-male transmission of genetically based non-obstructive azoospermia (NOA) and varicocele through a naturally obtained pregnancy.

**Subjects and methods:**

A father and his son were both diagnosed with NOA after centrifugation and varicocele. The father has no other clinical concerns apart from infertility, detected after many attempts of having another child, but given his urological situation (bilateral varicocele and NOA) assisted reproductive techniques were discouraged. After genetic counseling, several genetic-chromosomal analyses were carried out in the son (karyotype, chromosome Y microdeletions, *CFTR* screening, NGS infertility panels, and finally array-CGH).

**Results:**

After a series of inconclusive tests, array-CGH detected a deletion of 224–283 kb (del9p24.3) involving part of the *KANK1* and *DMRT1* genes, inherited from the father. Haploinsufficiency of *DMRT1* was therefore considered the determining factor in the development of azoospermia in the family by a loss of function mechanism.

**Conclusion:**

The confirmation of father-to-son transmission of a deletion including *DMRT1* represents an important point for clinicians dealing with male infertility, even when complete azoospermia is repetitively detected, and must be of hope for a relevant portion of men. Inclusion criteria for the access to assisted reproductive techniques may also be reconsidered and worthy of a greater number of clinical insights. Finally, since *DMRT1* alterations have been associated with NOA and abnormal testicular development, but not specifically with varicocele, further studies are required to validate this issue, as varicocele may have played a crucial role in this case.

## Introduction

Infertility, generally defined as failure to naturally achieve pregnancies after at least 1 year of attempts, affects approximately 10–15% of couples worldwide, and male factors are involved in about 50% of cases (https://www.who.int) [1–3]. Different causes of male infertility are known, including physiological, environmental, and genetic factors [4], and their identification is fundamental for effective management of infertile couples. Among such factors, one of the most significant is azoospermia, defined as the absence of sperm in the ejaculate. Azoospermia can be classified into obstructive and non-obstructive types, with non-obstructive azoospermia (NOA) often presenting a more complex diagnostic and therapeutic challenge, even because it affects about 10% of infertile men and 1% of general male population [5, 6].

NOA is generally due to intrinsic testicular failure in the spermatogenic process, with several possible underlying mechanisms making the etiopathology very heterogeneous [7, 8]. There are many known genetic causes underlying NOA, including chromosomal abnormalities and monogenic alterations, but to date over 70% of cases remain unexplained [9, 10].

One of the genes known to have a key role in NOA is *DMRT1*, which encodes a transcription factor involved in sex determination and gonadal development [11, 12]. Human chromosome 9 sub-telomeric deletions involving the *DMRT1* locus have been associated with 46,XY sex reversal and gonadal dysgenesis in XY individuals [13], while smaller deletions and sequence variants of *DMRT1* have been identified in men with phenotypes ranging from syndromic XY gonadal dysgenesis to azoospermia without signs of gonadal dysgenesis [14–20]. Therefore, although not yet indicated as a disease-associated gene in the OMIM database (https://www.omim.org), *DMRT1* is often included in molecular analysis aimed at identifying the genetic causes of NOA in infertile men.

In this report, we describe a case of male-to-male transmission of *DMRT1*-related NOA through a naturally obtained pregnancy, a more unique than rare case that can give hope to infertile couples and healthcare professionals involved in their clinical management.

## Case report

Patients discussed below had provided written informed consent to the use of their/their children’s biological samples and clinical data for research purposes.

A 14-year-old boy came to genetic counseling for personal and family history of azoospermia. He was recently diagnosed with 3^rd^-degree left varicocele. No significant structural alterations were identified by testicular ultrasound, whereas modest ectasia of the left pampiniform venous plexus and slight dilatation of the right testicular venous plexus in morpho-volumetrically normal testes and a homogeneous echo-structure without focal lesions were detected through scrotal Doppler ultrasound. Hormonal dosages documented increased levels of LH (9.3 UI/L) with normal levels of FSH (6.7 UI/L). He performed two spermiograms, both of which reported complete azoospermia even after centrifugation. After corrective surgery for varicocele, the absence of spermatozoa was confirmed on three biopsy samples obtained through Testicular Sperm Extraction (TESE). The boy is otherwise healthy, with no other clinical issues except for Rosai-Dorfman disease [21] localized in head and neck regions, for which he underwent multiple lesion removals.

The proband’s pregnancy was achieved naturally after a year of attempts when the father was 35 years old. Subsequently, due to missed second pregnancy, the parents underwent ascertainments which led to the diagnosis of infertility in the father. In particular, testicular ultrasounds detected bilateral varicocele (described as between 2^nd^ and 3^rd^ degree) and a cyst in the head of the right epididymis, hormonal dosages documented increased levels of FSH (19 UI/L) and LH (9.9 UI/L), and two subsequent spermiograms both showed complete azoospermia even after centrifugation. Chromosomal analysis for suspected Klinefelter syndrome [22] revealed a normal male karyotype (46,XY). Due to these results, the couple was not considered for assisted reproductive techniques (ART) due to the very low likelihood of success.

At family history collection, the proband was the only child of non-consanguineous parents, and no cases of azoospermia/infertility other than that of his father were reported.

In order to try to identify a genetic cause of the NOA documented in the family, several genetic-chromosomal analyses were carried out in the son (our proband):

- Karyotype: normal male (46,XY)
- Search for chromosome Y microdeletions: absents
- *CFTR* gene screening: wild type (wt)
- Next-generation sequencing (NGS) analysis of a panel of genes associated with 46,XY disorders of sexual developments (*AMH*, *DHX37*, *AMHR2*, *AR*, *CBX2*, *CYP17A1*, *DHH*, *GATA4*, *HSD17B3*, *HSD3B2*, *LHCGR*, *MAMLD1*, *MAP3K1*, *NR5A1*, *POR*, *SRD5A2*, *SRY*, *WT1*): absence of pathogenic/likely pathogenic variants
- NGS analysis of another panel of genes associated with male infertility (*AR*, *CATSPER1*, *FSHB*, *FSHR*, *KLH10*, *INSL3*, *NR5A1*, *RXFP2*, *SRY*, *TEX11*, *TEX15*, *DMRT1*): absence of pathogenic/likely pathogenic variants
- Analysis of the *SLC29A3* gene, associated with Rosai-Dorfman disease with possible involvement of the urogenital tract (MIM # 602782): wt

**Fig. 1:**
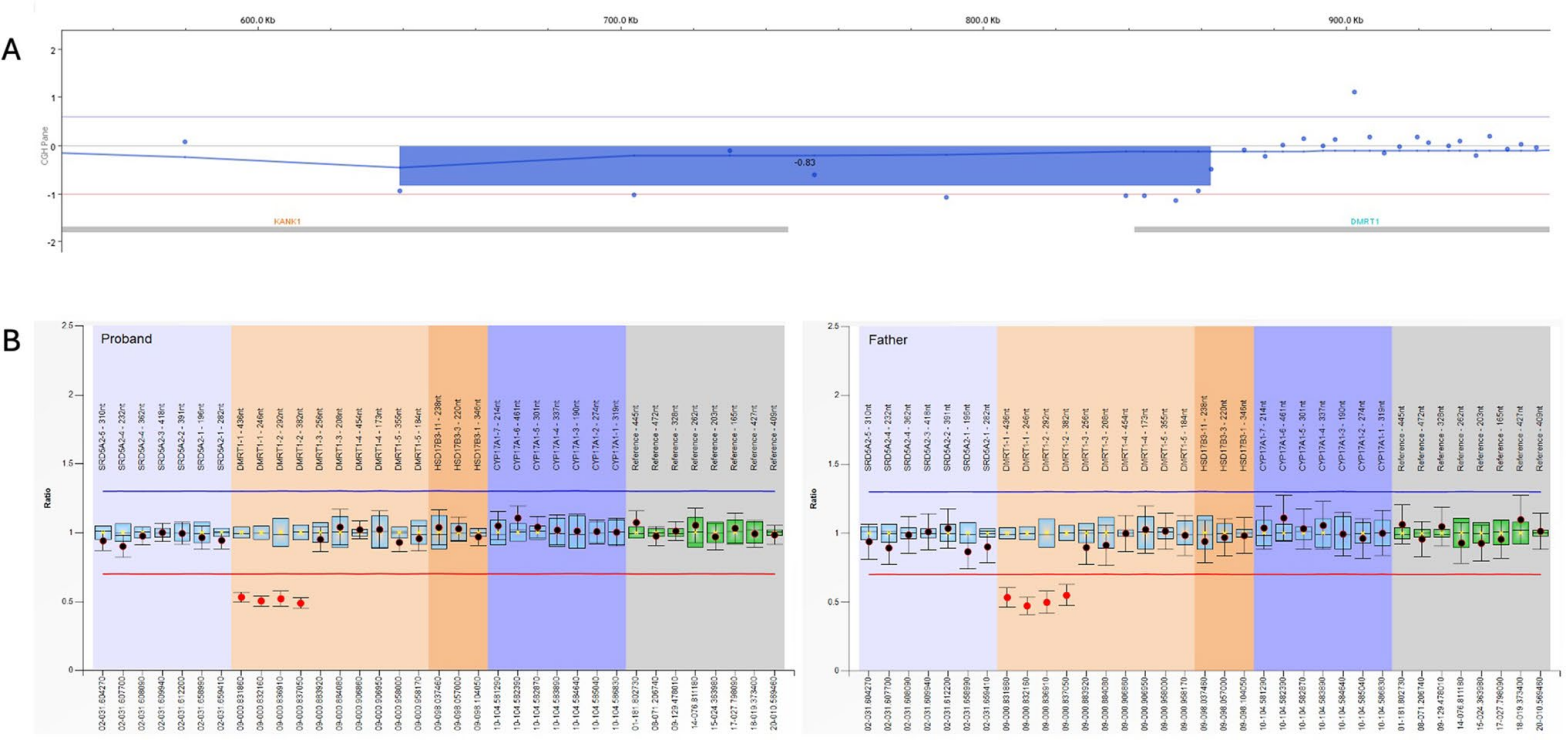
aCGH and MLPA results. **A** The aCGH profile of del9p24.3 identified in the proband, with the partial involvement of the *KANK1* and *DMRT1* genes. **B** The copy number ratios of the probes included in the MLPA probemix for both proband and father

Then, array-comparative genome hybridization (CGH) analysis was performed (Agilent 8 × 60 K ISCA platform with an average resolution of about 100 kb), with the detection of a deletion of 224–283 kb on the short arm of chromosome 9 (del9p24.3), involving part of the *KANK1* and *DMRT1* genes (Fig. 1A). Multiplex ligation-dependent probe amplification (MLPA - MRC Holland P334 Gonadal probemix) was then performed both in the son and in his parents, confirming the deletion of the first two exons of *DMRT1* and revealing father transmission (Fig. 1B).

Partial deletions encompassing the deleted region are present in the DGV database (http://dgv.tcag.ca/dgv/app/home) and are reported in ClinVar (https://www.ncbi.nlm.nih.gov/clinvar/) and DECIPHER (https://www.deciphergenomics.org) databases, where they are defined as of uncertain significance in most cases. Within OMIM database (https://www.omim.org), *KANK1 *deletions of paternal origin have been associated with cerebral palsy and spastic quadriplegia (MIM # 612,900), and subsequent work has suggested a possible role for gene deletions as susceptibility factors for neurodevelopmental disorders: however, a recent review suggests that small deletions involving *KANK1* should be considered benign [23]. Therefore, the haploinsufficiency of this gene is not to be implicated in the urological picture of our patients. Conversely, since chromosome 9 deletions involving *DMRT1* have been identified in patients with gonadal dysgenesis (https://clinicalgenome.org) and small deletions and single nucleotide variants of the gene have been detected in patients with azoospermia [10], the partial deletion of *DMRT1* is probably the determining factor in the development of azoospermia in the described family by a loss of function mechanism. The 9p24.3 deletion was therefore considered the underlying cause of the familial clinical picture.

## Discussion

Expanding knowledge on genetic causes and pathogenetic mechanisms that lead to NOA is crucial for the effective clinical management of infertile men. In this regard, the case described here provides new and useful elements on NOA and more specifically on conditions related to *DMRT1* gene alterations.

In fact, since the successful rate of pregnancy obtainment with ART, which include TESE to recover spermatozoa and intracytoplasmic sperm injection (ICSI) to fertilize oocytes, is significantly lower in cases of NOA than in cases of obstructive azoospermia [7, 24], the present case highlights how even in cases of complete NOA it is theoretically possible to naturally achieve pregnancies. To date, *DMRT1* alterations have been associated with NOA and abnormal testicular development, but not specifically with varicocele. In this family, both patients were diagnosed with NOA and varicocele at the same time: we can hypothesize that the latter is one of the manifestations associated with *DMRT1* alterations, and we can also speculate that varicocele may have played an additive role in the development of azoospermia. The father’s condition worsened after a spontaneous conception, so his clinical picture may have been initially less severe than what was highlighted later. In order to verify these hypotheses, it will be necessary to collect and evaluate a larger number of men with *DMRT1* alterations.

Regarding the role of the *DMRT1* gene, this case represents further evidence supporting its important role in male infertility, confirming the indication to include it among the genes to be investigated in cases of NOA of unexplained cause. However, the mechanisms that lead to the different phenotypes associated with gene alterations remain to be clarified: cases of azoospermia with no signs of gonadal dysgenesis and more complex pictures of syndromic XY gonadal dysgenesis have been described, without clear genotype–phenotype correlations [14–20]. In this regard, recently Emich and colleagues functionally evaluated different *DMRT1*variants and documented that variants localized in different domains of the DMRT1 protein are associated with different phenotypes and that the impact of the variant on the loss of protein function does not directly correlate with the severity of the phenotype [25]. Gonadal development is a very timely regulated process, and we can speculate that little imbalances between the DMRT1 protein and other interacting factors may cause differences in dysregulation which translates into relevant changes in phenotypes. Thus, further studies are necessary to better understand these mechanisms.

Anyway, the father-to-son transmission of del9p24.3 (and the consequent NOA with varicocele) which occurred in the family here described demonstrates that *DMRT1* alterations allow a residual conservation of a number of spermatozoa potentially sufficient to obtain pregnancies, even without ART, and this could also be valid for other overlapping conditions caused by similar molecular mechanisms. This once again highlights the fundamental importance of genetic diagnosis in infertile couples in order to personalize their clinical management and better predict their outcome. For example, in the light of this case, we now know that the presence of spermatozoa in men with *DMRT1* alterations is theoretically possible and therefore that these patients can hypothetically achieve natural pregnancies but above all they should not be excluded a priori from ART pathways.

More generally, the increasing availability of genetic testing is enabling the detection of previously undiagnosed causes of infertility. The accurate collection of clinical information on an ever-increasing number of patients with genetic infertility will lead to the possibility of a nearly personalized clinical management of patients, thereby improving the treatment outcomes.

## Conclusions

To the best of our knowledge, cases of naturally obtained pregnancies from patients with confirmed complete NOA are more unique than rare. Our case of father-to-son transmission of NOA and varicocele represents an exception to the rule, which can give hope for infertile couples and be a stimulus for development of increasingly effective medically ART. Once fully understanding the bio-pathological mechanisms that lead to testicular failure in these diseases, it will maybe be possible to increase the success rate of pregnancy obtainment naturally and/or through the improvement of ART.

## Data Availability

Individual patient data cannot be shared due to privacy or ethical restrictions. Requests for individual patient data can be submitted to the corresponding author.
